# The Lived Fracture Experience (LiFE) study: A mixed methods qualitative study research protocol exploring the lived experiences of fracture non-union patients

**DOI:** 10.1371/journal.pone.0318636

**Published:** 2025-04-08

**Authors:** Irene Yang, Carol Porteous, Hamish Simpson

**Affiliations:** 1 School of Informatics, Informatics Forum, Newington, Edinburgh, Scotland, United Kingdom; 2 Department of Orthopaedics and Trauma, University of Edinburgh, Edinburgh, Scotland, United Kingdom; 3 Department of Mechanical and Materials Engineering, Western University, London, Ontario, Canada; 4 Division of Orthopaedic Surgery, Department of Surgery, Schulich School of Medicine & Dentistry, Western University, London, Ontario, Canada; 5 Victoria Hospital, London Health Sciences Centre Research Institute, London, Ontario, Canada; 6 Usher Institute of Population Health Sciences and Informatics, University of Edinburgh, Edinburgh, Scotland, United Kingdom; 7 Edinburgh Orthopaedics, The Royal Infirmary of Edinburgh, Edinburgh, Scotland, United Kingdom; Southern Medical University Nanfang Hospital, CHINA

## Abstract

**Background:**

Fractures are a common and a major cause of disability and death, with up to 10% of fractures failing to heal normally. These fractures are called “fracture non-unions”. Currently, debate on the impact of non-union in the medical and research communities exists. Unfortunately, this indirectly minimizes the impact of fracture non-unions, and hinders further research work which could reduce unnecessary patient suffering. In this study, we aim to explore the lived experiences of fracture non-union patients in one-on-one interviews to determine whether fracture non-union is a burden for patients, and if so, to outline the burden on patients and their close circles.

**Methods:**

Eligible participants will include adult patients who currently have/previously (in the last 36 months) had a long bone fracture that has not healed normally. Data will be collected through in-depth face-to-face or telephone interviews, complemented by quantitative data captured using the standardized Euroqol EQ-5D-5L questionnaire. With consent, interviews will be audio-recorded and transcribed verbatim. Using NVIVO software, we will develop grouped codes and categories to describe and provide insight into the experience of fracture non-union patients. EQ-5D-5L, and EQ VAS data will be presented using a measure of central tendency and a measure of dispersion to provide insight into the overall health related quality of life for patients.

**Ethics and dissemination:**

The study was approved by the Health Research Authority and Health and Care Research Wales Approval (approval date: 27 March 2024; IRAS project ID: 337652). Results from this study aim to improve the information and support provided to fracture non-union patients. Findings will be disseminated to the study participants, healthcare professionals, and local commissioners through peer-reviewed articles and at academic conferences.

## Introduction

Fractures are a common and a major cause of disability and death worldwide: in 2019, an estimated 178 million people globally suffered from a fracture that warranted medical care [1]. Children and youths (<18 years old) are most at risk of fractures following sporting/physical activities or playing [2], whereas among the elderly population, most fractures are fragility fractures [3]. In 5–10% of all fractures, the fracture will fail to heal normally and these are known as fracture “non-unions” [4].

In addition to the global burden of fractures, fracture non-union, where the fracture has failed to heal, affects nine million patients/year worldwide [5], with numbers worsening with the ageing population. The impact of fracture non-union appears significant for the patient and the healthcare system: established non-union and even delayed union fractures are often complex to treat both surgically [6–9] and biologically [10,11], fixation techniques to treat non-unions [12] are associated with persistent poor clinical outcomes [13], and fracture non-union results in significant financial burden [14]. However despite this, debate on the impact of fracture non-union persists in the medical and research communities, which could be because no studies have explictly outlined the patient lived experiences of fracture non-union. This indirectly minimizes the impact of further research work relating to patients suffering from a fracture that fails to heal.

At the core of understanding the burden faced by patients, the outcomes that matter the most to patients, and how best to direct our research efforts, is the patient’s experience and perspective. In addition to quantitative tools, qualitative studies are an invaluable research tool that enables researchers to capture psychosocial, economic and emotional challenges faced by patients, and the outcome measures that matter the most to patients to build a comprehensive understanding of the full impact of a particular illness/condition such as fracture non-union. Aligning these burdens with research efforts and clinical care can provide sound rationale for research priority and funding, which can enhance the quality, relevance and applicability of research and the clinical care provided. Therefore, in this study, we aim to explore the lived experiences of non-union fracture patients through a mixed methods qualitative study, with the aim to determine whether or not fracture non-union is a burden for patients, and to outline the specific burden faced by patients.

## Methods

### Study design

**Fig 1:**
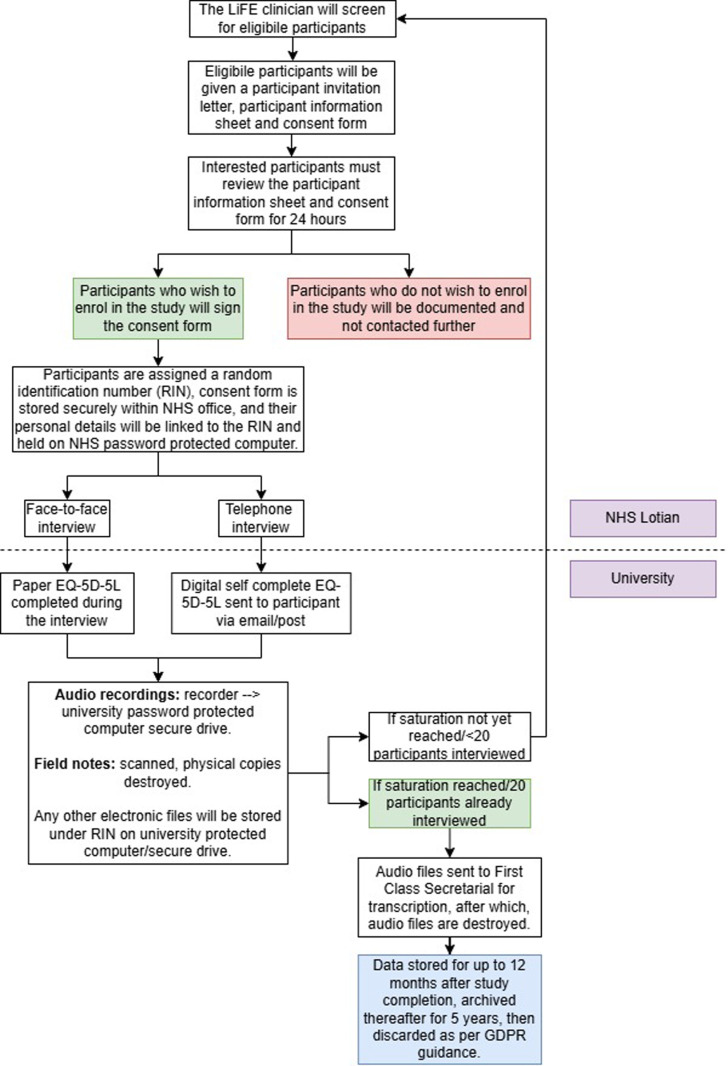
Study design flow chart. Dotted line shows whether the data will be held in NHS Lothian or within the university environment.

The study design is shown in Fig 1. The primary aim of the study is to explore the lived experiences of fracture non-union patients through a one-off, one-on-one, one hour interview with adult patients who currently have/previously (in the last 36 months) had a long bone fracture that has not healed normally. If in our study we find that having a fracture non-union is a burden for patients, the secondary aim is to provide greater insight into the burden it has on patients such as physical limitations, financial burden, mental health burden and to explore these effects on the patient as well as on their close family and friends.

### Research team

The LiFE Study research team is comprised the authors of this research protocol paper as well as members who have not been involved in the development of the research protocol. The current study team comprises two male clinician-researchers, including a professor of orthopaedic surgery and academic (HS) and a consultant orthopaedic surgeon and academic; one female clinician researcher, who is an orthopaedic registrar and academic; and five female researchers: 1) a biomedical scientist and academic research fellow in orthopaedic surgery (IY); 2) a medical student with a personal interest in orthopaedic surgery; 3) a research assistant/ orthopaedic research database manager with clinical research experience; 4) a clinical research nurse with experience as on an ethics committee panel; and 5) an expert patient and public involvement (PPI) lead with extensive qualitative research experience (CP). All team members either have first-hand experience of limb fractures or are closely related to someone who has experienced one.

### Study setting

The study is being conducted in the United Kingdom (UK) with participants (adult patients who currently have/previously (in the last 36 months) had a long bone fracture that has not healed normally, recruited from one clinical site in Scotland, The Royal Infirmary of Edinburgh (RIE). The RIE was chosen for this study as it is one of four major trauma centres in Scotland and part of the wider South East Trauma Network which provides care to people injured in the Lothians, Borders, Forth Valley and Fife, where people who have experienced major trauma injuries can access a consultant-led specialist team and receive appropriate diagnostic and treatment facilities for their major trauma. If in this study we find that fracture non-union patients face burden/s, this would provide us sound justification to conduct a larger scale study involving more major trauma centres to further explore this research question and solidify our understanding thereof.

### Participants and recruitment

Study inclusion and exclusion criteria are listed in Appendix I. To reduce recall bias, we aim to recruit adult patients who currently have a long bone fracture that is not healing normally. However, as we anticipate relatively low numbers of these patients, we have broadened the inclusion criteria to include adult patients who have previously (in the last 36 months) had a long bone fracture that has not healed normally. Potential study participants will be identified by clinical staff on fracture clinical lists. Once the clinician has identified eligible participants, the clinician will explain during in-person clinical consultations with the patient how they were identified, briefly outline the research study and its aim, and asked if they may be interested in taking part in the study. If the patient notifies the clinician that they are not interested, they will not be contacted any further. We aim to recruit up to maximum of twenty participants in the study, or until such time as there is “saturation” in the responses.

### Sample size

While no formal definition exists, saturation has become a normalised conceptual tool with consensus agreement that this approach is generally good practice in qualitative research [15]. Further, as fracture non-union affects 5-10% of fracture patients [14], our senior clinician (HS) anticipates low numbers of patients who would be eligible for enrolment in this study based on his extensive clinical experience with this patient cohort at the RIE. Therefore, in this study, we have suggested recruiting up to twenty patients as we think that this is a) feasible, b) should allow us to capture and the diversity of experiences and c) still enable manageable data analysis.

### Informed consent

If patients are interested in being a part of the research study, the clinician will give them a participant invitation letter, a copy of the patient information sheet (PIS) and the patient consent form (CF). The clinician may provide the patient with these documents directly, or ask the patient’s permission to be approached by a member of the research team to be given these documents. Eligible patients may also be sent the participant invitation letter, PIS and CF via mail. All eligible study participants will be asked to read and review the PIS and CF in full, for a minimum of 24 hours, and if they wish to take part/enrol in the study, participants will be required to return a signed CF to the clinician before the interview takes place to be assigned a random identification number. Participants who volunteer for telephone interviews meetings may post their consent form back using the enclosed prepaid envelope. We will obtain written informed consent from all study participants prior to interviewing them. The LiFE researcher conducting the interviews will confirm participant consent verbally at the start of the interview.

Recruitment commenced May 2024. At the time of writing, no participants have been recruited and participant recruitment and data collection is ongoing.

### Data collection

Data will be collected using a qualitative, semi-structured interview followed by a standardized questionnaire delivered by members of the LiFE study team. Participants will have the option for the interview to take place immediately after their next routine clinical consultation appointment. Alternatively, a telephone interview can be arranged, if preferred. As this is the first study to explore the lived experience of fracture non-union patients, the questions have been informed by Pryce et al. [16] and amended for our purposes based on the expertise of the authors. The questions have been designed to encompass the study participant’s motivations to take part in the study, the lived experience of fracture non-union patients, including the referral and diagnostic journeys/processes, the orthopaedic services and support, challenges of living with a fracture non-union, how study participants have managed their fracture non-union and finally the impact of the fracture non-union on their personal well-being and mental health and the impact on their family/close circle including financial impacts, any care responsibilities and personal relationships. We will use open-ended questions to facilitate and encourage the study participants to deeply explore their experiences with flexibility (Appendix II). We anticipate the interviews to take up to one hour with an additional 30 minutes allocated for study participants to complete the standardized Euroqol EQ-5D-5L questionnaire [17]. With participants’ permission, the interviews will be recorded using an encrypted digital recorder (Olympus DS9000), which means that the recording files are protected using an algorithm, and that the device itself is pin-locked to keep the information secure. The level of encryption is acceptable to National Health Service (NHS) Lothian (AES256). In addition to audio recordings, field notes will be taken to capture important information not already audio recorded.

Study participants will be interviewed by a member of the LiFE Study research team. If possible, it is our intention to have the same interviewer for all study participants to ensure consistency in data collection. At the start of each interview, the interviewers will discuss with the study participant their role as well as the researcher’s role in the study. Interview timing and the nature of the interview will also be discussed, which will include the researcher’s interest and motivations in the topic.

All transcripts will be anonymised, and pseudonyms will be used. We will not return transcripts to participants for review, including commenting and/or correction. This decision was made to enable us to best capture the experiences and views at the time of interview.

### Data analysis

#### Interviews.

In line with Strauss and Corbin’s grounded theory procedures [18,19], an iterative approach to data collection and analysis will be taken, with early data collected, analysed and interpreted and compared to inform ongoing interviews. To explain this further, interviewers will keep field notes during the interview to document any observations, thoughts and points worth reflecting on. These will be written out more clearly immediately following each interview to ensure accuracy and clarity in the data capturing process. The wider LiFE Study research team will meet regularly to discuss the field notes. To ensure that we are capturing a wide range of views and experiences with enough depth of understanding to provide useful insights that enable us to address our research aims, we will continue to review the data until we are confident that a wide range of experiences have been captured. Using NVivo software (Lumivero LLC, USA), each line of the interview transcript will undergo open coding by one member of the LiFE Study research team independently to summarise key content, and identify initial themes, which will be reviewed and amended by the wider research team. Codes and themes will be developed through examining the interview transcripts only, to allow an inductive and exploratory analysis. Codes will be compared across and between transcripts and collated into broader categories to capture the range of experiences for analysis and interpretation of the data by the wider research team. We will consider any core categories that arise that may offer insight into the experiences of living with a fracture non-union and explain variation within the data set. Our analysis will aim to describe the range of ‘burden’ of living with a fracture non-union including well-being, mental health, financial and personal impacts on patients and their close circles.

#### EQ-5D-5L questionnaire.

While various tools exist to measure health and health related quality of life [20], the EQ-5D is well-established tool recommended by the National Institute for Health and Care Excellence (NICE), and is the most commonly used generic measure for assessing health-related quality of life in the health sector [21]. Therefore, in our study, we decided to use the latest version of the EQ-5D questionnaire, the EQ-5D-5L, which was designed to address the ceiling effect seen with the original EQ-5D questionnaire [20]. Our choice of the EQ-5D-5L questionnaire over other existing health and health related quality of life measure tools was based on the simplicity of the tool, enabling easier and quicker data collection from participants, which was especially important given that this data would be collected in addition to the one hour qualitative interviews. The EQ-5D-5L captures some information on the overall health related quality of life, which enables calculation of cost-effectiveness for health economics evaluations that are standardised across populations suitable for international studies, which may be particularly relevant in subsequent studies that we hope to conduct.

As per the EQ-5D-5L questionnaire results guidance [22], we will describe the number and percentage of patients reporting each level of problem on each dimension of the EQ-5D-5L. EQ VAS data and the EQ-5D-5L index data will be presented using a measure of central tendency and a measure of dispersion. Depending on the data and if it is skewed, this could be the mean value and the standard deviation if the data are not skewed or, the median values and the interquartile range if the data are skewed.

#### Rigor in analysis.

We aim to be transparent about our decision making throughout the research study undertaking to ensure research credibility. The study team will meet regularly to discuss, compare and contrast their experiences to identify and address any assumptions or oversights held by the individual researchers within the LiFE study team, and to help shape data collection and data interpretation. Throughout the study, the wider LiFE research group will discuss interpretations of the findings and work together to develop and link theoretical categories for data analysis. The lead researcher will also discuss transcript coding with CP, to leverage their expertise in qualitative data analysis. The lead investigators will supervise the analysis closely to ensure consistency throughout the research process.

### Ethics and dissemination

#### Ethical approval.

This study was reviewed and approved by the Health Research Authority and Health and Care Research Wales Approval (approval date: 27 March 2024; IRAS project ID: 337652). The study also received approval from the National Health Service (NHS)’s Research and Development (R&D) group on the 2 May 2024.

#### Participant withdrawal.

Each participant has the right to withdraw at any point within four weeks following the interview, without having to justify their decision. All withdrawals will be recorded accordingly. If the participant decides to withdraw from the study, any information that they have given will be destroyed and they will not be contacted by the LiFE research team again.

#### Confidentiality.

All data obtained from participants will be kept confidential. Participants will be anonymised with all identifying information removed. Participants’ names will be replaced with pseudonyms in the transcripts.

#### Benefits to participation.

Participants may share and reflect on their experience of living with a fracture with the LiFE Study research team, which they may find empowering as they may feel that their experiences are heard/listened to. Their insights will help inform our understanding of the impact of fracture non-union, so that we can justify research efforts to improve the care for patients with a fracture non-union.

#### Assessment and management of risks.

There is a small possibility that answering some of the questions, or discussing certain topics will create distress for study participants. If a participant experiences distress at any point during the interview, they can ask the interviewer to pause the recording. If needed, we can stop the interview. If study participants experience distress after the interview is completed, the researcher will sign post them to NHS 24 mental health services, which include listening, offering advice, and guiding them to further help if necessary. The NHS 24 mental health services are available to everyone in Scotland 24 hours a day, 7 days of the week.

#### Storage of data.

Audio recordings from the interview will be saved under the random identification number and transferred using a password protected laptop/computer owned by the research team from the recording device to a University of Edinburgh managed DataStore service which is managed by the university and with access limited to members of the LiFE Study research team only. For each audio recording, after the interview has concluded and a minimum of four weeks after the interview has passed, audio recordings will be sent for transcription. Following transcription of audio files, the audio recording files will be appropriately destroyed by deleting the files.

During the interview, the interviewer will record the participant’s basic personal details (name, CHI number, DOB/age, sex, ethnicity, address, telephone number and email address) which will link each study participant with their assigned random identification number. To ensure retention of personal identifiable information within NHS Lothian, this file along with the patient consent form will not leave NHS Lothian and will be stored on an NHS password protected computer.

Also during the interview, details about the study participant’s fracture (when the fracture was sustained and how) and questions that pertain to the impact that the fracture non-union has had on their life, their wellbeing and on their family/close circle may be recorded by the interviewer in a separate excel spreadsheet. This excel spreadsheet will not contain any participant’s personal information, only their random identification number and will be stored separate to the master excel spreadsheet and participant consent forms on a university password protected computer to ensure participant confidentiality. Data collection will be carried out using a password protected laptop owned by the research team, data will be uploaded onto university managed server which only the study team will have access to.

If any hand written notes are made during the interview, in accordance with the University of Edinburgh Research Data Service recommendations, all electronic data will be stored on a password-protected computer file and any paper records will be promptly destroyed once electronic copies have been made and securely stored.

All electronic data will be stored on a password-protected computer file and any paper records will be promptly destroyed once electronic copies have been made and securely stored. Once the completion of the structured interview, study participants will also be asked to complete an EQ-5D-5L questionnaire to supplement the qualitative study with a measurable metric. Other than the participant’s responses to the questions, EQ-5D-5L questionnaire will also not contain any participant’s personal information, just their random identification number, and will be stored on a university password protected computer.

#### Dissemination of research findings.

We will prepare and publish articles in appropriate peer-reviewed journals and professional magazines to ensure the findings are shared with orthopaedic healthcare professionals and academics. Findings will be disseminated via poster and oral presentations at relevant academic conferences, and social media. Articles will also be disseminated to study participants who opt in for these updates, and will form the basis for development of future PPI groups, orthopaedic services and local commissioners.

## Appendix I

Inclusion criteria:

1. Adult patients (≥ 18 years of age)
2. Currently have/previously (in the last 36 months) had a fracture that has not healed normally
3. Able to provide informed consent
4. English speakers.

Exclusion criteria:

1. Patients (< 18 years of age).
2. Patients with no history of a fracture that has not healed normally.
3. Unable to provide informed consent
4. Non – English speakers

## Appendix II

1. Tell me a little bit about yourself?
2. Tell me your story with when and how you sustained your fracture non-union and why you were interested in taking part in this study?
3. Can you tell me about the treatment and care
  a. How did/do you feel about the care that you have received since your fracture non-union?
4. How did having this fracture non-union impact your daily life?
5. What have you found difficult during the healing of your fracture non-union?
6. What have you done to manage your fracture non-union?
7. Can you please describe the impact that the fracture non-union has had on your well-being and mental health?
8. Can you please describe the impact that the fracture non-union has had on you and your family?
  a. Financial
  b. Care responsibilities
  c. Personal relationships
